# Superconducting State Properties of CuBa_2_Ca_3_Cu_4_O_10+δ_

**DOI:** 10.3390/ma16145111

**Published:** 2023-07-20

**Authors:** Artem Lynnyk, Roman Puzniak, Luchuan Shi, Jianfa Zhao, Changqing Jin

**Affiliations:** 1 Institute of Physics, Polish Academy of Sciences, Aleja Lotników 32/46, PL-02668 Warsaw, Poland; puzni@ifpan.edu.pl; 2 Beijing National Laboratory for Condensed Matter Physics, Institute of Physics, Chinese Academy of Sciences, Beijing 100190, China; 3 Songshan Lake Materials Laboratory, Dongguan 523808, China

**Keywords:** high-*T*_c_ superconductors, superconducting state properties, critical current density, grain boundaries, magnetic studies

## Abstract

The superconducting state properties of the CuBa_2_Ca_3_Cu_4_O_10+δ_ (Cu-1234) system, with a transition temperature as high as 117.5 K, were investigated. The ac magnetic susceptibility measurements confirmed a very sharp transition to the superconducting state. The upper critical field, *H*_c2_, as high as 91 T, and the irreversibility field, *H*_irr_, as high as 21 T at 77 K, were determined using dc SQUID magnetization measurements. The intragrain critical current density, *j*_c_, estimated from a magnetic hysteresis loop, is as high as 5 × 10^9^ A/m^2^ in a self-generated magnetic field at 77 K. However, the intergrain critical current density in the studied material is smaller by four orders of magnitude due to very weak intergrain connections.

## 1. Introduction

In more than three decades since the discovery of high-*T*_c_ superconductivity in 1986 [1], type II superconducting cuprates are considered more than highly promising from both scientific and practical points of view. The possible usability of YBa_2_Cu_3_O_7-δ_ (YBaCuO, Y-123) [2], Bi_2_Sr_2_Ca*_n_*_-1_Cu*_n_*O_2*n*+4+δ_ (BiSrCaCuO, Bi-22(*n*-1)*n*) [3,4], HgBa_2_Ca*_n_*_-1_Cu*_n_*O_2*n*+2+δ_ (HgBaCaCuO, Hg-12(*n*-1)*n*) [5,6], and Tl_2_Ba_2_Ca*_n_*_-1_Cu*_n_*O_2*n*+4+δ_ (TlBaCaCuO, Tl-12(*n*-1)*n*) [7,8] was already proven and verified in high-power applications: SMES, power cables, etc. [9]. The symbol *n* in the above formulas is an integer number, which indicates the exact amount of specific chemical elements in the unit cell, hence the stoichiometry of the material. The symbol δ is a decimal number, which lies within the (0, 1) interval and indicates the deviation of the oxygen amount from the stoichiometric content.

However, YBaCuO possesses a critical temperature, *T*_c_ ≈ 93 K, that is only slightly higher than liquid nitrogen’s boiling temperature (77 K). Meanwhile, high anisotropy of the superconducting parameters of BiSrCaCuO [10] quite complicates its utilization in mass-production [11], and both HgBaCaCuO [12] and TlBaCaCuO [13] contain toxic materials.

The high-pressure and high-temperature synthesized CuBa_2_Ca_3_Cu_4_O_10+δ_ (Cu-1234) system is intensively studied, because of its relatively high superconducting transition temperature *T*_c_ of about 116 K at the ambient pressure together with its low crystallographic anisotropy and lack of toxic elements [14,15,16], which are crucial if we have to deal with the utilization of this material in devices. The above mentioned facts, as well as a high value of the critical current density *j*_c_ of the order of 10^4^ A/cm^2^ at 77 K [17], make the Cu-1234 system a cut above other groups in the cuprate family of high-temperature superconductors (HTSCs) from the device-production perspective. However, the fact that real conductors, i.e., wires, cables, etc., should be polycrystalline emphasizes an extra significance of the consideration of structural imperfections (e.g., grain boundaries), whose presence, in turn, strongly influences the superconducting state parameters of a material [18,19]. The role of the irreversibility field *H*_irr_(*T*) in the limitations of the critical current also can not be underestimated since that field points out the entrance of the material to a flux-creep regime. That is why chasing the improvement of superconducting parameters demands the defining of phase diagrams of the upper critical field *H*_c2_(*T*), *H*_irr_(*T*), and the lower critical field *H*_c1_(*T*), which, together with microscopic parameters’ coherence length *ξ* and penetration depth *λ*, well enough describe the superconducting nature of the probed system.

In the present work, the superconducting state properties of polycrystalline CuBa_2_Ca_3_Cu_4_O_10+*δ*_ are investigated with the SQUID magnetometry technique by means of both direct current (dc) and alternating current (ac) methods. It is shown that the upper critical field, *H*_c2_, of the studied material is as high as 91 T, and the irreversibility field, *H*_irr_, is as high as 21 T at 77 K. Furthermore, the intragrain critical current density, *j*_c_, estimated from a magnetic hysteresis loop, is as high as 5 × 10^9^ A/m^2^ in a self-generated magnetic field at 77 K. Hence, the CuBa_2_Ca_3_Cu_4_O_10+δ_ (Cu-1234) material seems to be highly promising from the perspective of high-power applications.

## 2. Materials and Methods

The solid state reaction under high-pressure and high-temperature was applied for the obtained Cu-1234 specimen [16,20,21,22]. The comprehensive experimental procedure’s description can be found in the relevant source [20]. CuO (Alfa Aesar, Ward Hill, Massachusetts, USA, 99.995% purity), CaO (Alfa Aesar, 99.95% purity), and BaO_2_ (Alfa Aesar, 95% purity) were employed as the initial components. All procedures were carried out in an argon-filled glove box to maintain oxygen and moisture levels below 1 ppm, since alkaline earth metallic oxides exhibit high hygroscopicity. The high-pressure treatment was conducted using a cubic anvil-type apparatus. The sample was gradually subjected to a pressure of 6 GPa and subsequently heated to 1273 K, and then it was maintained at this temperature for a duration of 30 min. After that, the temperature was lowered to an ambient level before pressure reducing. The utilization of high-pressure and high-temperature conditions is considered as optimal to obtain the stable uniform perovskite phase of the target material.

The neutron powder diffraction (NPD) technique was utilized for rigorous crystal structure investigations. It turned out that the samples of Cu-1234 synthesized in exactly the same way possessed a tetragonal crystal structure of the P4/mmm space group with the following lattice parameters: *a* = *b* = 3.85856(5) Å, *c* = 17.9544(6) Å (No. 123) [17]. It was established that the 1234 phase found in the Hg-based (e.g., HgBa_2_Ca_3_Cu_4_O_10+δ_ [23]) or Tl-based (e.g., TlBa_2_Ca_3_Cu_4_O_10+δ_ [24]) systems closely resembles the currently investigated Cu-1234 sample from the crystal structure perspective [22]. The NPD refinement revealed that the precise chemical composition of the Cu-1234 sample is Cu_0.94_Ba_2_Ca_3_Cu_4_O_10.66_, which de facto means that the equality of an average valence state of Cu is up to +2.29 [17]. The structural components [Ba_2_CuO_3+δ_] and [Ca_3_Cu_4_O_8_] play the roles of charge reservoir blocks (CRBs) and superconducting blocks (SCBs), respectively. The hole doping level was quantitatively estimated by means of O-*K* XAS [25,26,27]. Meanwhile, the transitions from O 1*s* to the doped hole states are related to the first peak of the spectrum, and the upper Hubbard band corresponds to the second one. The over-doped nature of Cu-1234 [17] was defined by comparing the spectral weight of the first peak recorded for Cu-1234 and observed in YBa_2_Cu^2.30+^_3_O_6.95_ [28], which turned out to be stronger with regard to Cu-1234, suggesting a higher doping level in Cu-1234. Considering Ba_2_Cu^2.40+^O_3.2_ [29,30,31], the first peak is lower than in the Cu-1234 spectrum; hence, Ba_2_Cu^2.40+^O_3.2_ possesses a higher doping level with regard to Cu-1234. The X-ray diffraction (XRD) experiments for the Cu-1234 material were performed in the work [20] and confirmed the type of crystal structure of the currently investigated sample. The lattice parameters were in good consistency with those presented above, i.e., *a* = *b* = 3.85 Å, *c* = 18.30 Å.

The morphology of CuBa_2_Ca_3_Cu_4_O_10+δ_ and the related microcrystalline structure in this material were investigated by means of the STEM (scanning transmission electron microscopy, Hillsboro, Oregon, USA) technique in the work [17]. The results indicate the existence of plate-like 90º microdomains of a size up to 50 nm × 500 nm along the transverse and longitudinal components, respectively. The Raman spectrum of (Cu*_x_*C_1-*x*_)Ba_2_Ca_3_Cu_4_O*_y_* was measured by Y. Tanaka et al. [32] revealed that two successive anomalies with a decreasing temperature were attributed to two superconducting gaps having different magnitudes.

The Quantum Design Magnetic Property Measurement System (MPMS) *XL* SQUID was utilized in order to study the superconducting state properties of the presented material. Depending on the purpose, either direct current (dc) or alternating current (ac) methods were applied with high precision to the order of 10^−8^ emu provided in both cases. In order to protect the clearness of the experiments, the investigated sample was fixed inside a non-magnetic standard plastic straw, which gave a homogeneous background during the measurements. By applying the dc method, both the temperature and field dependencies of the magnetic moment were recorded. The temperature dependencies of the magnetic moment were obtained by means of zero-field cooling (ZFC) and field-cooled cooling (FCC) protocols. The ZFC protocol consisted of measurements of the system at warming after cooling at a zero external magnetic field, while FCC comprised measurements at cooling right after ZFC. The magnetic moment, as a function of the dc magnetic field, was measured at fixed temperatures after cooling at zero field. Ac susceptibility in the form of simultaneously recorded real *χ*′ (in-phase component) and imaginary *χ*″ (out-of-phase component) parts was gauged by exploiting the ZFC protocol at a fixed frequency of the oscillating ac field without the application of a superimposed dc magnetic field.

## 3. Results and Discussion

### 3.1. Ac Susceptibility and Transition to Superconducting State

**Figure 1:**
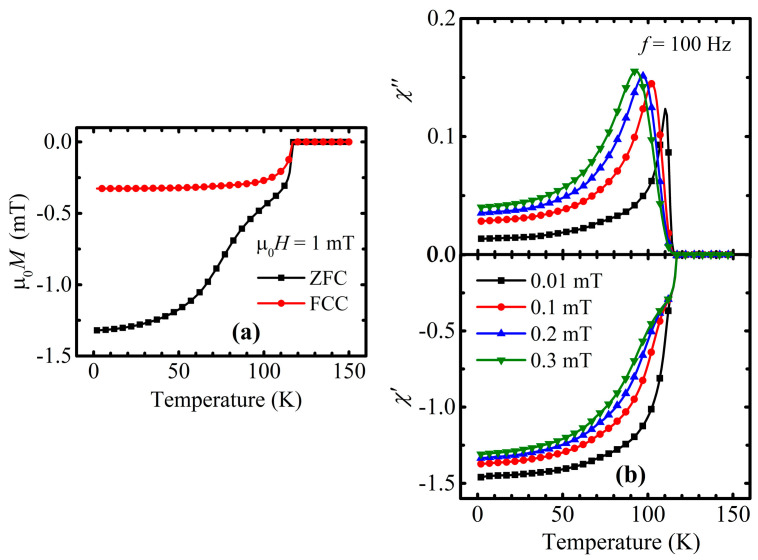
(**a**) Temperature dependence of dc magnetization recorded at 1 mT of external field in zero-field cooling (ZFC) and field-cooling (FCC) modes. (**b**) Temperature dependence of an imaginary part *χ*″ and a real part *χ*′ of ac susceptibility (ac magnetization normalized by corresponding ac field value) measured with an ac field of 100 Hz frequency and with the following amplitudes: 0.01 mT (black squares), 0.1 mT (red circles), 0.2 mT (blue triangles), and 0.3 mT (green inverted triangles). Recorded susceptibility value extends diamagnetic limit because presented data are not corrected for demagnetizing field.

The preliminary results were obtained in the form of temperature dependencies of the magnetic moment at a fixed dc field of 1 mT (Figure 1a). The initial analysis of the presented curves revealed the onset of a transition to the superconducting state at a temperature *T*_c_^onset^ as high as 117.5 K, which is comparable to the literature data [16,17] for materials from the Cu-1234 group but with different oxygen contents. A value of the superconducting volume fraction of about 100% was estimated from the ZFC magnetization recorded at 2 K by considering the demagnetization effect. The bifurcated character of the ZFC–FCC curves is usually associated with a certain level of vortex matter pinning [33,34,35]. The presence of a second phase transition was defined from the ZFC dependence in the vicinity of 100 K and can be explained as either the co-existence of a secondary superconducting phase [20,36] or as a step-like weak links behavior [37,38], which very often happens in similar granular materials.

In order to probe the superconducting state of the system more thoroughly, ac susceptibility was measured. Technically, measurements were conducted by applying oscillating fields with the amplitudes of 0.01, 0.1, 0.2, and 0.3 mT sequentially—one ac field for each of the curves. Meanwhile, the frequency of 100 Hz was common for all of the ac susceptibility experiments. The results, shown in Figure 1b, confirmed the validity of the dc investigations and the correctness of the *T*_c_^onset^ estimated value as well as the unambiguity of the second transition occurrence. While the onset points of the diamagnetic behavior on the in-phase component *χ*′ remains static and the sharpness is not affected by the strength of the applied ac field (Figure 1b), the step-like transition becomes more pronounced and shifts to lower temperatures, with the ac field increasing. It is worth noting that the magnitude of the saturation values of *χ*′ at a low temperature only weakly depends on the magnetic field, indicating the improvement of the response from the shielding phase. Simultaneously, the corresponding out-of-phase loss peak *χ*″ (Figure 1b) increases its amplitude value and repeats the movement of the bend on the real part component. A general pattern of the obtained ac curves is similar to the results which were described for YBaCuO in [39,40] and corresponded to a weak links behavior.

Regarding the weak links theory, granular systems are considered as bunches of grains which are connected to each other through grain boundaries, i.e., Josephson junctions [41]. It is established that defective grain boundaries possess rather poor superconducting properties and cannot, as effectively, transfer the supercurrent as grains itself [42,43,44]. That is why the presence of such weak junctions causes a non-simultaneous response to the applied external magnetic field. According to the above written, the onset of the superconducting behavior revealed on *χ*′(*T*) is the consequence of the circulation of screening currents on the grains’ surfaces—intragranular currents. Meanwhile, the current through weak connections—intergranular—starts to contribute to screening of the material as a whole only on cooling below the step-like transition. With this background, the single loss peak character of *χ*″(*T*) strongly confirmed the inhomogeneous nature of the investigated sample, since it correlates with the intergranular step-like transition.

**Figure 2:**
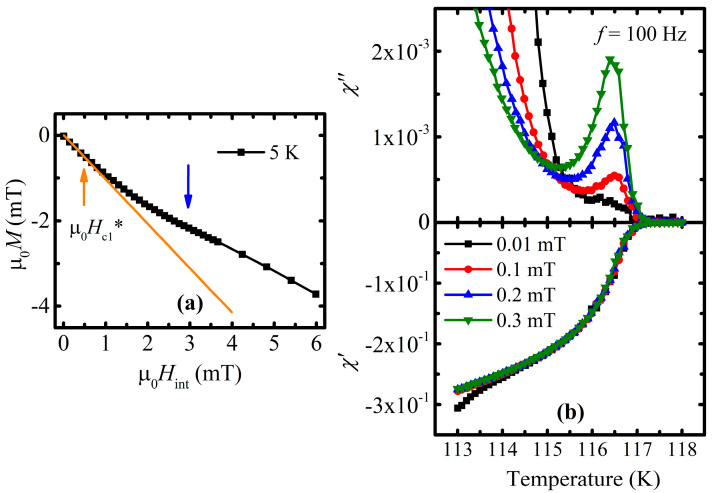
(**a**) The initial magnetization curve as a function of the µ_0_*H*_int_ magnetic field at a temperature of 5 K. The blue arrow points out the bend, which reflects the transition from a lower-field intergranular to higher-field intragranular regime. Orange arrow indicates the penetration of the material with supercurrent vortices within the intergranular regime. (**b**) The imaginary and real parts of ac susceptibility, presented in Figure 1b, are redrawn in the narrow range of the temperature in the vicinity of the transition to superconducting state; *χ*″(*T*) depicts the dynamics of the intragranular loss peak change.

A more careful analysis of the ac susceptibility revealed the existence of the additional loss peak right in the vicinity of the superconducting state’s onset (Figure 2b), which comes from the penetration of the magnetic field into the grains [40,45,46]. This means that the huge loss peak in Figure 1b completely originates from the dissipation which is connected with the circulation of the screening current through weak junctions.

The initial magnetization curve was obtained within the small range of the external dc field at 5 K and is represented as a function of the internal field µ_0_*H*_int_ along the procedure of the demagnetizing effect correction: *H*_int_ = *H*_ext_—*DM* (*H*_ext_—applied dc magnetic field, *M—*measured magnetization, *D*—demagnetizing factor) (Figure 2a). A fitted Meissner line (orange line in Figure 2a) was utilized for the experimental estimation of the demagnetizing factor *D*, which turned out to be equal to 0.27 and is valid for all the results in the present work, since the sample was measured in the same direction with regard to the applied external dc field.

The dependence itself clearly indicates the presence of the bend. Apparently, the inflection corresponds to the transfer from intergranular to intragranular screening at higher fields [47]. Similar to [47], there can be the supposed existence of an intergranular lower critical field µ_0_*H*_c1_^*^(5 K) as large as 0.5 mT. Since the bend appears at a relatively small field of about 3 mT, the following high-field magnetization experiments were carried out within the intragranular regime, i.e., where the grains behave independently.

### 3.2. Superconducting State Parameters—Upper and Lower Critical Fields

**Figure 3:**
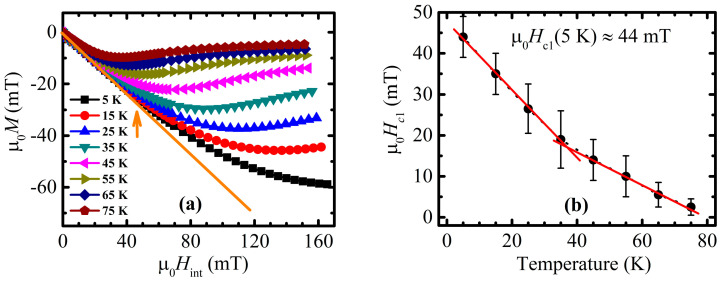
(**a**) Isothermal field dependencies of magnetization. Orange arrow points to the moment of deviation of the 5 K magnetization curve from the ideal intragranular diamagnetic response (orange straight line). (**b**) Temperature dependence of lower critical field, determined as a point of magnetization deviation from linear intragranular diamagnetic response for the various temperatures given in panel (**a**). Presented error bars determine the accuracy of estimation of the field of detectable deviation appearance.

The isothermal field dependence of the magnetization was measured in an internal magnetic field of up to 170 mT at various temperatures in the range from 5 K to 75 K for the purpose of the lower critical field *H*_c1_(*T*) determination (Figure 3a). One may assume that deviation from the linear diamagnetic behavior, reconstructed above low-field bending, matches with the beginning of the penetration of the separated grains with Abrikosov vortices [48,49,50]. The points corresponding to deviation from the zero-magnetization value were determined following the subtraction of the low-field diamagnetic straight line of the separated grains (orange line on Figure 3a), and the phase diagram of *H*_c1_(*T*) was constructed (Figure 3b). Generally, in the case of granular material with weakly coupled grains, one can distinguish between two well-separated deviations from two straight lines: (i) the first one is from the line defining the diamagnetic response of the full bulk sample, as seen in Figure 2b, corresponding to the intergranular lower critical field, *H*_c1_*; and (ii) the second one is from the line defining the diamagnetic response of separated grains, as seen in Figure 3a, corresponding to the intragranular lower critical field, *H*_c1_.

However, it is important to notice that intragranular *H*_c1_(*T*), defined from the initial isothermal magnetization curves, is highly vulnerable to the influence of grain boundaries. The relatively inferior shielding abilities of grain boundaries and their low crystal quality create a high possibility of penetration of that area with the magnetic field, with subsequent pinning. Since this can happen earlier than on the grains themselves, the whole magnetic response from the material during measurements can be distorted. As a consequence, the values of *H*_c1_(*T*) defined by that approach can be underestimated. This is why *H*_c1_(*T*) dependence defined from high-field *λ* value measurements, discussed later on in this subchapter, is supposed to be more reliable.

The general appearance of *H*_c1_(*T*) dependence, presented in Figure 3b, turned out to be irregular and can roughly be divided into two parts: low and high temperature, with the inflection point in the vicinity of 15 mT (see, Figure 3b). Such behavior strongly supports supposition of the underestimation of *H*_c1_(T), determined with the beginning of the penetration of the separated grains with Abrikosov vortices, especially in higher temperatures. The lower critical field at 5 K, estimated in the way described above, is equal to 44 mT.

**Figure 4:**
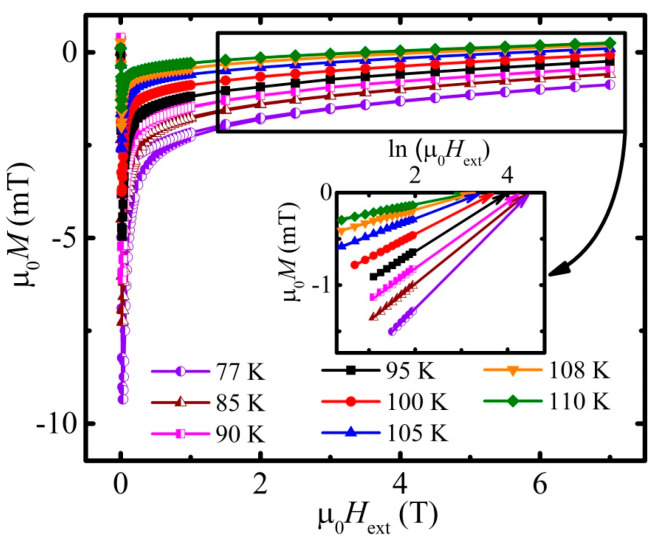
Field dependence of magnetization for half of hysteresis loop at the following temperatures: 77 K (violet half-filled circles), 85 K (brown half-filled triangles), 90 K (magenta half-filled squares), 95 K (black squares), 100 K (red circles), 105 K (blue triangles), 108 K (orange inverted triangles), and 110 K (green rhombs). Inset illustrates determination of an upper critical field *H*_c2_ from logarithmic field dependence of the reversible part of magnetization. Arrows represent the linear extrapolation of the µ_0_*M* vs. ln(µ_0_*H*_ext_) dependence to the zero-magnetization value. Legend on the main panel is valid also for the inset.

**Figure 5:**
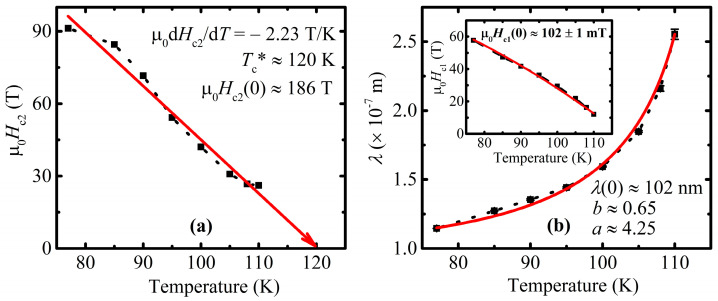
(**a**) An upper critical field as a function of temperature. Red arrow points the critical temperature *T*_c_* predicted by Werthamer–Helfand–Hohenberg (WHH) theory. The calculated parameters of the WHH equation, i.e., critical temperature *T*_c_*, slope –µ_0_d*H*_c2_/d*T*, and zero-temperature upper critical field µ_0_*H*_c2_ (0), are placed directly in the figure. (**b**) Estimated values of *λ*(*T*) and *H*_c1_(*T*) (inset) are subsequently fitted (solid red line); fitting parameters are depicted immediately in corresponding panels.

The field dependencies of magnetization in the form of isothermal loops were measured, aiming at studying the irreversible properties of the material as well as for obtaining upper critical field and penetration depth (see Figure 4 and Figure 5, respectively). The conspicuous thing about those loops is their absolute narrow shape, i.e., ascending and descending branches of the loops are close to each other (main panel in Figure 4), which hints at rather poor irreversible properties and, as a consequence, at a relatively low value of the bulk’s (transport) critical current density, *j*_c_*.

The upper critical field µ_0_*H*_c2_ and the penetration depth *λ* in turn were defined from the same field dependencies of magnetization, but by considering the initial magnetization curve, which is practically almost merged with the fully reversible part of it, i.e., where the loop is completely closed—the region is embraced with a black rectangle on the main panel. The idea of *H*_c2_ and *λ* estimation is based on the London approach of the 2nd type superconductors (SCs) magnetizing scheme. For high-*T*_c_ superconductors, there exists a broad field domain where *H*_c1_ << *H* << *H*_c2_. Within such a range of field, the reversible magnetization *M* is known to be linearly proportional to ln*H* [51,52] such that:

(1) $$M\left(H\right)=-\frac{\Phi_{0}}{8\pi \mu_{0}\lambda^{2}}\left(\ln\frac{{\eta H}_{c2}}{H}\right)$$

and the *H*_c2_ in fact lays in extrapolation of the linear dependence of the high field magnetization till its intersection with the logarithmic dependence of the magnetic field axis. Parameter *η* in Equation (1) is a parameter of the order of unity, *λ*—field penetration depth.

The inset in Figure 4 illustrates a set of linear tails of the hysteresis loops, which were plotted in a semi-logarithmic scale. In order to eliminate the influence of possible paramagnetic inclusions, a considered set of curves was obtained after the subtraction of the corresponding Curie and Van Vleck paramagnetic contributions, which in turn were calculated separately for each of the measured dependencies.

The approximation and subsequent extrapolation of the resulting curves yielded upon reaching the intersection with the field axis and indicates a destroying of the superconductivity in the material, i.e., obtaining *H*_c2_ for a specific temperature. The gathered points in turn formed the dependence of µ_0_*H*_c2_(*T*), which is shown in Figure 5a for the investigated Cu-1234 sample. It is important to admit that due to the specificity of the extrapolation process itself, the low-temperature points of *H*_c2_(*T*) are estimated less accurately simply because of a greater distance between the considered and measured low-temperature curve and its desired intersection with the field axis. Nevertheless, the experimentally estimated errors for all of the obtained *H*_c2_(*T*) points lay within the depicted symbols in Figure 5a in a temperature range above 77 K.

Concurrently, it should be noticed that factor $\frac{\Phi_{0}}{8\pi \mu_{0}\lambda^{2}}$ in Equation (1) is responsible for the slope of *M*(ln*H*) dependencies (inset on Figure 4). Hence, the field penetration depth *λ* was assessed from *M*(ln*H*). The obtained data for *λ*(*T*) are presented in the main panel of Figure 5b.

In order to estimate the upper critical field at a zero temperature *H*_c2_(0), the Werthamer–Helfand–Hohenberg (WHH) equation in the dirty limit approach was applied [53,54,55]:

(2) $${{µ}_{0}H}_{c2}\left(0\right)=-0.693\left(\frac{{{µ}_{0}dH}_{c2}}{dT}\right)T_{c}^{*}$$

The value of the transition temperature *T*_c_* predicted by the WHH theory was defined from the linear approximation of the µ_0_*H*_c2_(*T*) diagram (red arrow on Figure 5) and equaled to 120 K, which is 2.5 K higher than the measured *T*_c_^onset^, indicating that the temperature dependence of *H*_c2_ in the close vicinity of *T*_c_ is stronger than the linear one, and the upper critical field of the studied system does not show features characteristic of two-band superconductivity. The slope of the arrow gave the –µ_0_d*H*_c2_/d*T* a parameter equal to 2.23 T/K, which is quite high for high-*T*_c_ superconductors.

The coherence length *ξ* was calculated by utilizing the Ginzburg–Landau expression [56], which links the upper critical field and coherence length:

(3) $${{µ}_{0}H}_{c2}=\frac{\Phi_{0}}{2\pi \xi^{2}}\;.$$

The temperature dependence of *λ*(*T*) was approximated by phenomenological dependence [56]:

(4) $$\lambda \left(T\right)=\lambda_{0}/{\left[1-{\left(\frac{T}{T_{c}}\right)}^{a}\right]}^{b},$$

with the three fitting parameters *λ*_0_, *a*, and *b* given in Figure 5b. The obtained values are close to the two-fluid model parameters, where *a* = 4, *b* = 0.5.

The following equation [56]

(5) $${{µ}_{0}H}_{c1}=\frac{\Phi_{0}}{4\pi \lambda^{2}}\left[ln\left(\frac{\lambda }{\xi }\right)+0.5\right]$$

was used for the calculation of the lower critical field *H*_c1_(*T*) values, which are related to the high-field *M*(ln*H*_ext_) measurements (Figure 4). The *H*_c1_(*T*) dependence itself, depicted in the inset of Figure 5b, was approximated by means of the following relation [56]:

(6) $${{µ}_{0}H}_{c1}={{µ}_{0}H}_{c1}\left(0\right)\left[1-{\left(\frac{T}{T_{c}}\right)}^{2}\right].$$

Table 1 represents the summarized superconducting state parameters of the investigated system. The studied Cu-1234 is characterized by a very large Ginzburg–Landau parameter value equal to 77, a very large zero temperature upper critical field value equal to 186 T, and a very small coherence length of 1.33 nm, which indicate a high pinning efficiency of small-point defects that may be intentionally introduced to the studied material.

### 3.3. Irreversibility Line and Intragrain Critical Current Density

**Figure 6:**
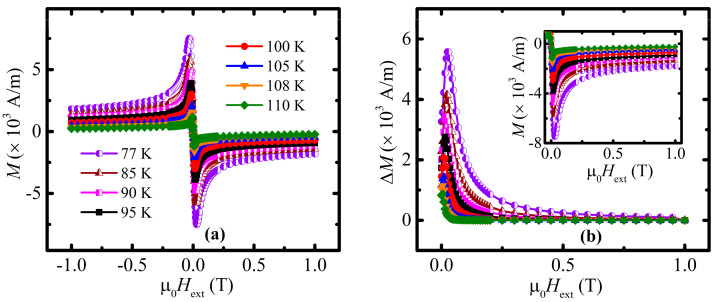
(**a**) Field dependence of magnetization at the following temperatures: 77 K (violet half-filled circles), 85 K (brown half-filled triangles), 90 K (magenta half-filled squares), 95 K (black squares), 100 K (red circles), 105 K (blue triangles), 108 K (orange inverted triangles), 110 K (green rhombs), and 112 K (violet stars). (**b**) Field dependence of Δ*M* obtained for the corresponding temperatures. Inset in Figure 6b shows the data, which were immediately used for the Δ*M*(µ_0_*H*_ext_) curves’ calculation.

Necessary magnetization hysteresis loops in the limited field range of [−1; 1] T (Figure 6a) were measured and are represented in A/m units in order to obtain the critical current density *j*_c_ from the perspective of Bean’s critical state model. For each of isothermal curve, the corresponding parameter Δ*M*(µ_0_*H*_ext_) was calculated (main panel on Figure 6b) as a difference between the ascending and descending branches of the field dependencies of magnetization (inset on Figure 6b).

According to the critical state model [57], the density of the critical current can be estimated from the width of the hysteresis loop Δ*M*, expressed as [58]:

(7) $$j_{c}=\frac{2\Delta M}{d}.$$

In the case of the homogenous bulk superconductors, parameter *d* is related to the geometrical size of the investigated sample. In the case of granular superconductors in a high magnetic field, i.e., where the grains are decoupled, parameter *d* should be treated as an average grain size. Since the hysteresis loops of the studied material were recorded in relatively high fields (see the previous considerations related to *H*_c1_ defining), the recorded width of the hysteresis is related to the intragranular critical current, and thus, for the assumed approximately spherical shape of an average grain, *d* = 2*c*, where *c* is a radius of the grain. In the case of granular superconductors, with the grain radius *c* comparable with the penetration depth *λ*, the shielding of the external magnetic field is not complete even in the field below *H*_c1_, and the measured magnetic susceptibility χ in the field below *H*_c1_ is expressed by [59]:

(8) $$\frac{\chi }{\chi_{max}}=1-3\frac{\lambda }{c}\coth\frac{c}{\lambda }+3\frac{\lambda^{2}}{c^{2}}\;,$$

where *χ_max_* describes the intergranular susceptibility of the dense bulk superconductor in the magnetic field below *H*_c1_*. Taking into account the experimental data presented in Figure 2a and Figure 3a, corrected for the demagnetizing field, one can obtain $\frac{\chi }{\chi_{max}}=0.588$. Thus, for the studied material with a zero temperature *λ*(0 K) = 102 nm, the estimated average radius of the grains *c* is equal to 621 nm.

**Figure 7:**
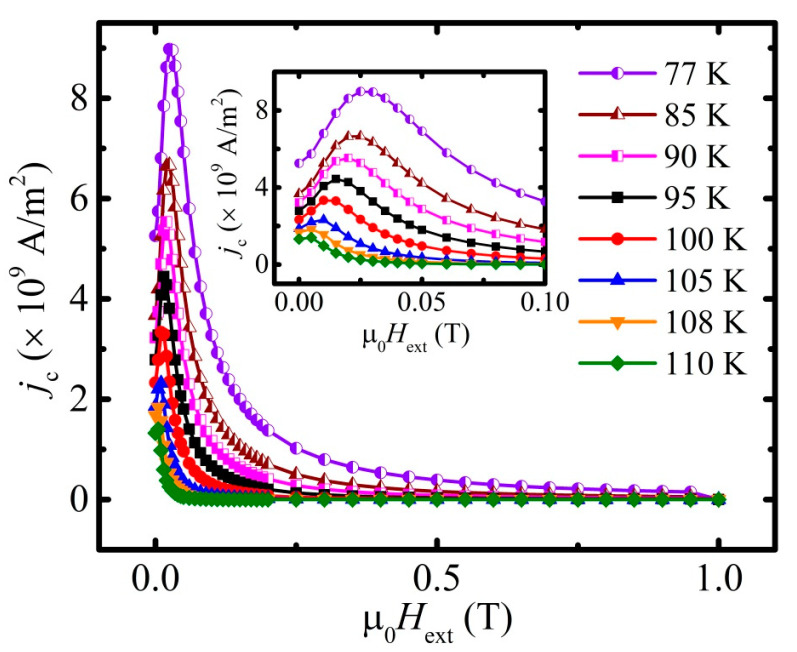
Field dependence of *j*_c_ obtained for the corresponding temperatures: 77 K (violet half-filled circles), 85 K (brown half-filled triangles), 90 K (magenta half-filled squares), 95 K (black squares), 100 K (red circles), 105 K (blue triangles), 108 K (orange inverted triangles), 110 K (green rhombs), and 112 K (violet stars). Inset points out the presence of maximum on *j*_c_(µ_0_*H*_ext_) curves.

**Figure 8:**
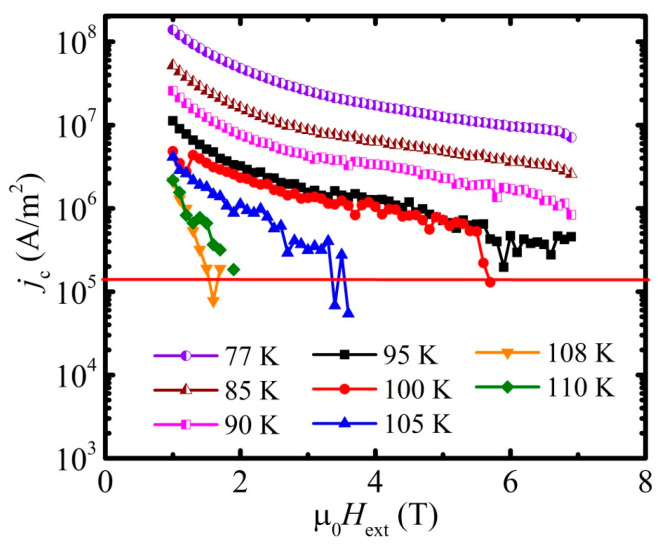
Field dependence of *j*_c_ calculated within the range of 1–7 T of the external magnetic field µ_0_*H*_ext_ and represented in a logarithmic scale. The legend is the same as for the previous figure.

The critical current density *j*_c_, calculated with Equation (7) with an average grain size *d* = 2*c* equal to 1242 nm, applied for the width of the hysteresis loop given in Figure 6b, is presented in Figure 7. The same approach applied for the data given in Figure 4 leads to the data presented in Figure 8.

**Figure 9:**
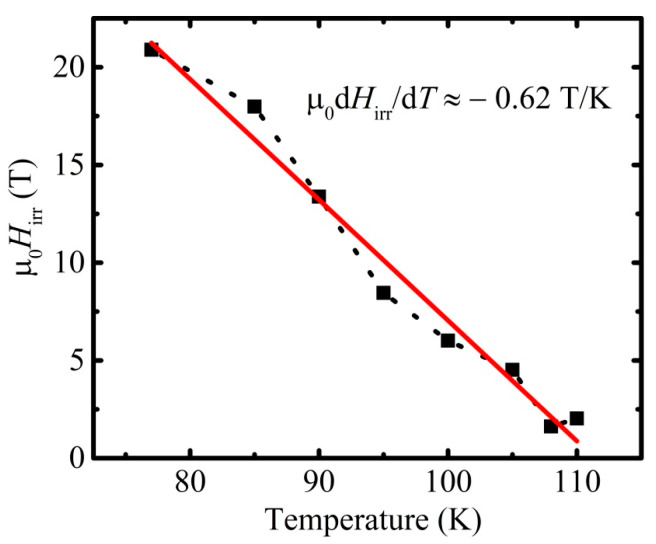
The irreversibility field line, calculated from high-field dependencies of *j*_c_(µ_0_*H*_ext_). The linear approximation (red line) of the dependence and its slope are depicted.

With the assumption for the criterion of an appearance of irreversibility as high as *j*_c_ = 1.5 × 10^5^ A/m^2^, which excludes the overestimation of the obtained results, one can obtain the irreversibility field line presented in Figure 9 with the irreversibility field, *H*_irr_, at 77 K as high as 21 T. The data for the temperatures below 100 K were obtained by a cross of the linear extrapolation of the log*j*_c_(*H*) dependence with the line of the constant value *j*_c_(*H*) = 1.5 × 10^5^ A/m^2^.

### 3.4. Limitations of Material Application—Intergrain Critical Current Density

The intergrain critical current density was estimated based on the position of the low-temperature peak in the imaginary part of the ac susceptibility, presented in Figure 1b. The applied procedure was described by D.-X. Chen et al. [60]. The procedure was developed assuming a non-uniform distribution of the magnetic field within the granular material. With respect to the proposed approach, the intergranular critical current density $j_{c}^{*}$ was calculated according to the following relation:

(9) $$j_{c}^{*}=\frac{H_{p}}{x}.$$

Parameter *H*_p_ corresponds to the full penetration field and approximately equals to the amplitude of the applied ac field—*H*_ac_. Since the assumption is valid for the samples of the square cross-section in the out-of-plane direction with regard to the applied field, parameter *x* was considered as a half of the geometrical size of the investigated sample in the out-of-plane direction. The obtained data are presented in Table 2.

The linear extrapolation of the data presented in Table 2 to the lower temperature range leads to the value of an intergranular critical current density of about 2.9 × 10^5^ A/m^2^ at 77 K, which is smaller by four orders of magnitude than the intragranular critical current density at that temperature. This is most likely due to the weak links caused by the presence of impurity phases in the studied material [61]. Being non-superconducting, such phases can break the intergranular connections, deteriorate screening abilities, and, as a consequence, strongly limit the values of the transport’s critical current density.

## 4. Conclusions

It was shown that the upper critical field *H*_c2_ of CuBa_2_Ca_3_Cu_4_O_10+δ_, with a transition temperature as high as 117.5 K, is as high as 91 T at 77 K, and the irreversibility field *H*_irr_ at 77 K is as high as 21 T. Furthermore, the intragrain critical current density, *j*_c_, estimated from the magnetic hysteresis loop, is as high as 5 × 10^9^ A/m^2^ in a self-generated magnetic field at 77 K. However, the intergrain critical current density in the studied material is smaller by four orders of magnitude due to the very weak intergrain connections. It is supposed that the cause of the weak links’ existence probably lies in the presence of impurity phases. Being non-superconducting, such phases can break the intergranular connections, deteriorate screening abilities, and, as a consequence, strongly limit the values of the critical current. Nevertheless, the relatively high values of *H*_c2_ obtained in the intragranular regime indicate a high quality of the major target phase, i.e., CuBa_2_Ca_3_Cu_4_O_10+δ_.

## Figures and Tables

**Table 1:** Measured and calculated superconducting state parameters of the Cu-1234 system. The second row of the table depicts superconducting state parameters of YBa_2_Cu_3_O_7-δ_ for the sake of comparison.

Material	*T*_c_^onset^ (K)	*λ*(0) (nm)	µ_0_*H*_c1_(0) (mT)	µ_0_d*H*_c2_/d*T* (T/K)	µ_0_*H*_c2_(0) (T)	*ξ*(0) (nm)	µ_0_*H*_c2_(77K) (T)
Cu-1234	117.5	102	102	–2.23	186	1.33	91
Y-123	92.2	89	90	−1.9	122	1.64	25

**Table 2:** The values of intergrain critical current density $j_{c}^{*}$, calculated from ac susceptibility measurements. *T*_m_—temperature, which corresponds to the maximum on temperature dependence of the out-of-phase component of the ac susceptibility. *H*_p_, in that particular case, is considered to be equal to the applied ac field *H*_ac_. The average geometrical size of the sample in the perpendicular to the applied ac field direction: 2*x* = 4 × 10^−3^ m.

*H*_p_ (A/m)	*T*_m_ (K)	$j_{c}^{*}$ (A/m^2^)
8	110.5	4000
80	103	40,000
160	98	80,000
240	94	120,000

## Data Availability

The data that support the findings of this study are available from the corresponding author upon reasonable request.
